# Diffusion MR Imaging of the Brain in Patients with Cancer

**DOI:** 10.1155/2011/714021

**Published:** 2011-10-25

**Authors:** J. Matthew Debnam, Dawid Schellingerhout

**Affiliations:** Department of Radiology, Section of Neuroradiology, The University of Texas MD Anderson Cancer Center, 1400 Pressler Street, FCT16.024, Unit 1482, Houston, TX 77030, USA

## Abstract

Over the last several years, there has been significant advancement in the molecular characterization of intracranial diseases, particularly cerebral neoplasms. While nuclear medicine technology, including PET/CT, has been at the foreground of exploration, new MR imaging techniques, specifically diffusion-weighted and diffusion tensor imaging, have shown interesting applications towards advancing our understanding of cancer involving the brain. In this paper, we review the fundamentals and basic physics of these techniques, and their applications to patient care for both general diagnostic use and in answering specific questions in selection of patients in terms of expected response to treatment.

## 1. Diffusion MR Imaging: Fundamentals and Physics

Diffusion magnetic resonance imaging (MR) utilizes the Brownian motion of molecules to derive images. Robert Brown first described the motion bearing his name in 1827 [1] after observing the random motion of pollen grains suspended in water. Movement caused by the random movements of water molecules has endlessly fascinated scientists, even Einstein, who in 1907 speculated that the speed of the particles subject to Brownian motion would be dependent only on temperature, and not on the size and nature of the particle or on its environment [2]. Einstein speculated that this prediction would be impossible to verify experimentally due to the technical challenges involved in taking measurements on extremely short time scales and microscopic spatial scales. Recent work though, using laser tweezers to measure a silicon dioxide microsphere's motion in air, has measured instantaneous velocities of the particle following a Maxwell-Boltzmann distribution; a mean square displacement velocity of 0.422 mm/s has been determined [3]. However, the technical demands for such measurements in water rather than air are still beyond current technical capabilities, confirming Einstein's prediction after more than a century. This primal kinetic activity of fluid molecules is responsible for the diffusion and intermixing of fluids and dissolved substances and is the process targeted for measurement with diffusion MR imaging. Through carefully designed MR sequences, much can be learned about the diffusion of water molecules, and by implication, the structure of the organ or substrate the water is in. 

In MR imaging, the local magnetic field determines the rate of precession, or wobbling, of hydrogen nuclei. With diffusion MR imaging, a gradient is applied and precession is induced by an applied radiofrequency pulse. The gradient is then reversed and an equal and opposite radiofrequency pulse applied. The purpose of this is to “null” or decrease the signal from the stationary protons, while the mobile protons, which have changed position during time period between the two radiofrequency pulses, will not be nulled perfectly. 

Measuring the signal of the mobile protons allows determination of the amount of diffusion which has occurred, and this is represented as a mathematical equation. By rearranging this mathematical equation to isolate the diffusion coefficient, information about diffusion within each voxel can be obtained. The magnitude of diffusion within the voxel is measured by the Apparent Diffusion Coefficient (ADC), and this information can be used to create an image called an ADC map. A low value for ADC indicates that molecular movement in the sampled tissue is restricted (dark signal), while a high value for ADC indicates that the tissue has free diffusion (bright signal). 

### 1.1. Application: Stroke

Some of the earliest applications of diffusion-weighted imaging (DWI) in humans were to diagnose cerebral infarction [4]. It was found that ADC values dropped quickly after infarction, with a nadir reached at about 24 hours, and that infarcts could be diagnosed earlier on the DWI sequence than on T2-weighted images. Diffusion imaging rapidly became the new standard of imaging care in stroke and was soon augmented by perfusion-based imaging strategies [5].

The mechanism of why stroke shows restricted diffusion of water is still unclear, but is thought to be due to ischemia causing failure of the ionic pumps which usually maintain ion gradients across the cell membrane; water then diffuses into the cells, which become swollen. This net shift of water from the extracellular environment, where it can diffuse relatively freely, to the intracellular environment, where diffusion is relatively restricted, is thought to be the mechanism for restricted diffusion. It also explains the transient nature of the ADC abnormality, as the cytotoxic cells eventually lyse; this rupture and destruction of organized matrix again lets water molecules diffuse freely. ADC typically returns to normal as the infarct evolves to encephalomalacia [6].

**Figure 1:**
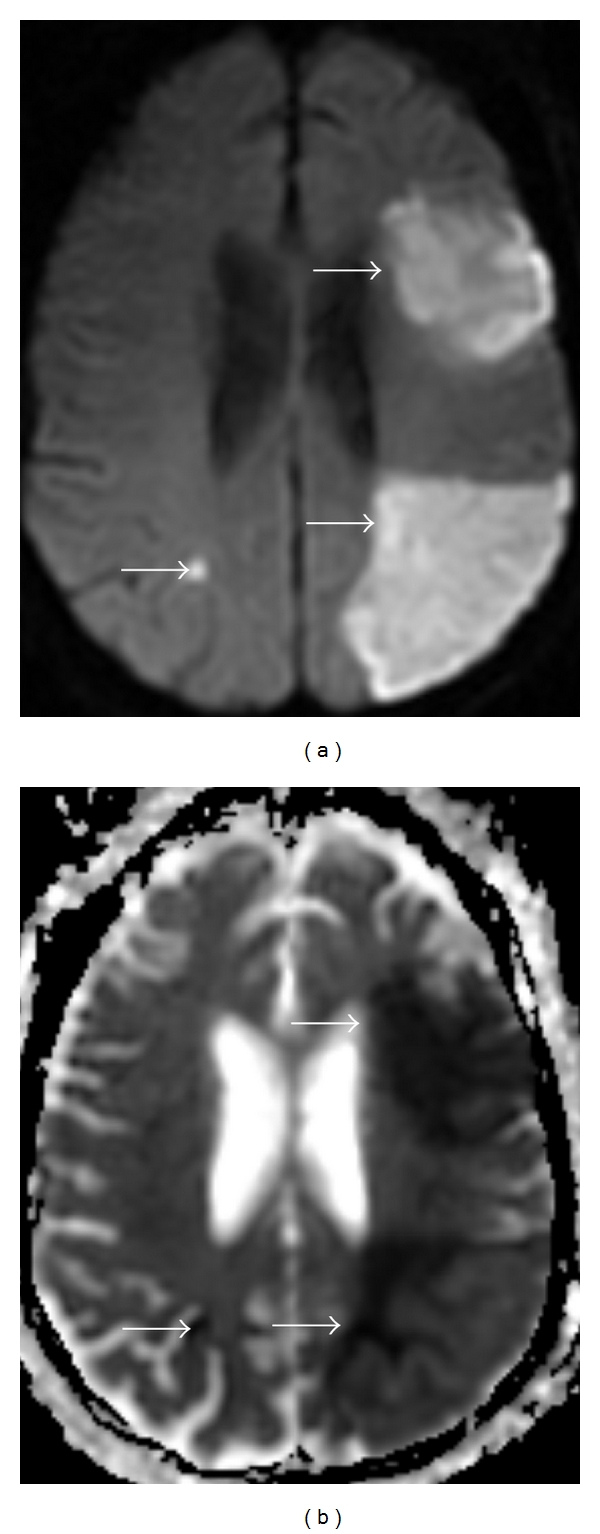
A 76-year-old female with history of acute myelogenous leukemia and atrial fibrillation, presenting with altered mental status related to embolic stroke. (a) DWI sequence. (b) ADC map. Areas of signal hyperintensity on the DWI sequence (arrows) are confirmed to represent restricted diffusion by the signal hypointensity on the ADC map (arrows).

When evaluating for acute ischemic stroke, one first interprets the trace DWI sequence, looking for signal hyperintensity. A limitation of the DWI sequence is that it is sensitive, but not specific for the detection of restricted diffusion. If there is signal hyperintensity present on a routine T2-weighted images, such as from cytotoxic edema, this signal hyperintensity may be present on the DWI sequence, an effect referred to as “*T2 shine-through*”. To determine if the signal hyperintensity seen on the DWI sequence truly represents decreased diffusion, an ADC sequence is reviewed. The ADC sequence is not as sensitive as the DWI sequence for restricted diffusion, but is more specific, as the ADC images are not susceptible to the “*T2 shine-through*” effect. Ischemic brain parenchyma will show a low ADC value and associated edema will not. Therefore, an acute stroke will have signal hyperintensity on the DWI sequence and low signal intensity on ADC sequence (Figure 1).

### 1.2. Application: Diffusion Imaging in Abscess and Tumors

**Figure 2:**
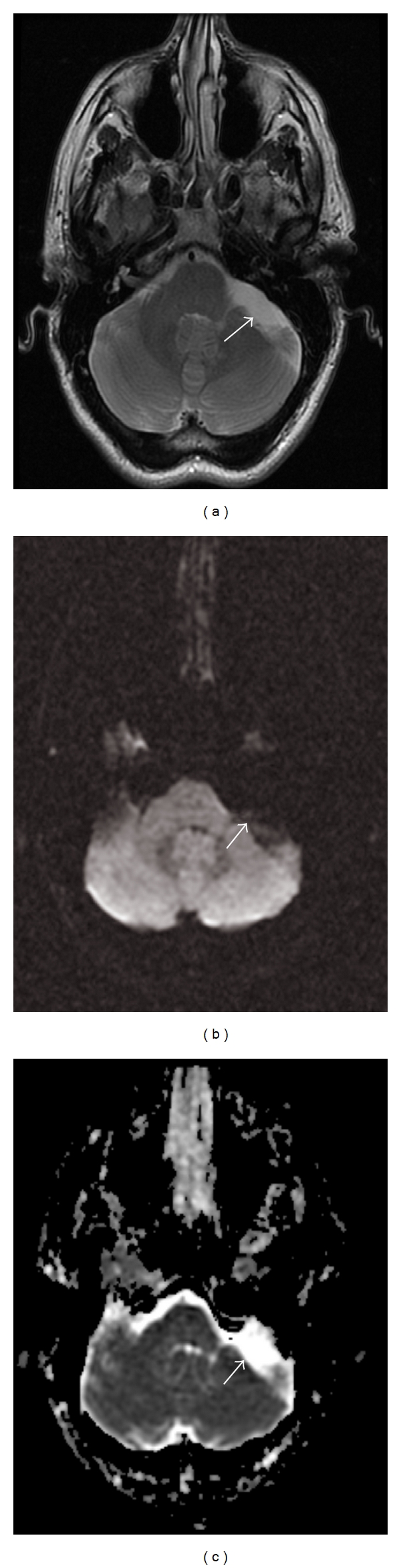
A 32-year-old man with a metastatic melanoma with an incidentally detected left cerebellopontine angle arachnoid cyst. (a) Axial Fast Spin Echo T2 sequence. T2 hyperintensity is seen in the cerebellopontine angle mass (arrow). (b) DWI sequence. The mass is dark on diffusion-weighted imaging. (c) ADC map. Signal hyperintensity in the lesion on the ADC map (arrow) is present. These findings are consistent with a cyst and exclude other lesions such as an epidermoid.

**Figure 3:**
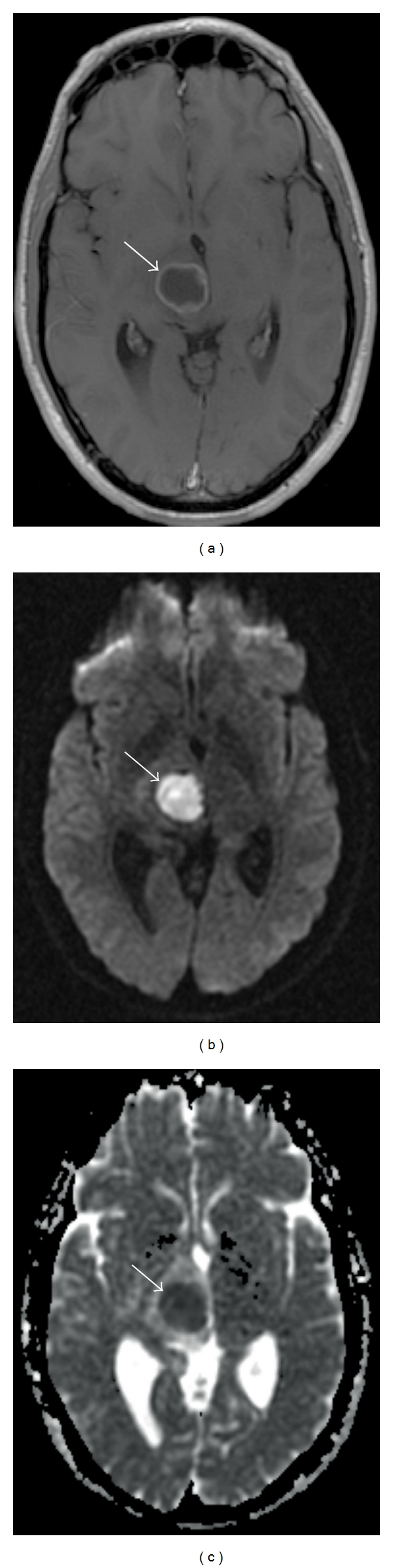
A 19-year-old man presented with diplopia and left-sided weakness, and a right thalamic abscess. (a) Axial T1 postcontrast. A ring enhancing mass is present in the right thalamus (arrow). (b) DWI sequence. Signal hyperintensity is present in the lesion (arrow). (c) ADC map. Signal hypointensity in the lesion (arrow) confirms decreased diffusion. In the appropriate clinical context, this constellation of findings is consistent with a pyogenic abscess.

As diffusion-weighted imaging provides information about the mobility of water molecules in, for example, brain parenchyma, tumors, pus, and cysts (Figure 2), it can be classified as a type of molecular imaging [7]. Ebisu et al. [8] first reported high signal intensity of abscess pus on the DWI sequence with a corresponding low ADC value consistent with restricted diffusion (Figure 3). 

 Given that cell death seems to induce restricted diffusion in stroke, there has been much interest in utilizing this principle for imaging cancer, where cell death of the malignancy is the desired therapeutic outcome. Several authors [9, 10] have described that tumor cellularity is a major determinate of ADC values in brain tumors. Solid tumors are generally more water-rich than normal brain and are thus brighter on T2-weighted images and have a higher ADC. Surrounding edema, unfortunately shares these same attributes, making the DWI sequence less valuable in the distinction of tumor borders. 

**Figure 4:**
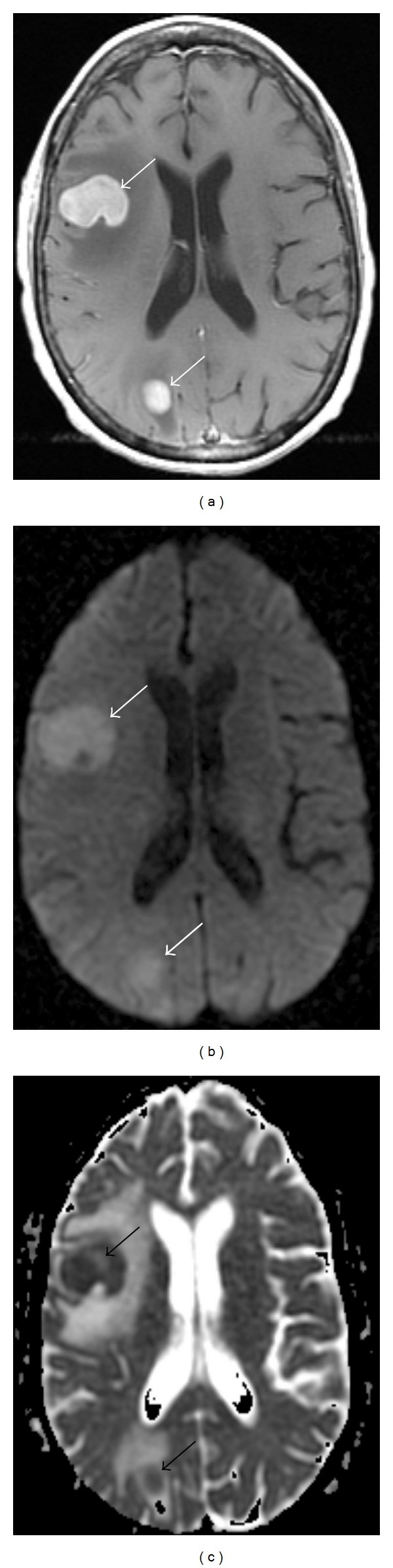
A 63-year-old female with diffuse large B-cell lymphoma involving the frontal and parietal lobes. (a) Axial T1 postcontrast. Diffusely enhancing right cerebral mass lesions are present (white arrows). (b) DWI sequence. The lesions are hyperintense on diffusion-weighted imaging (white arrows). (c) ADC map. The ADC map shows hypointensity of the lesions (dark arrows).

**Figure 5:**
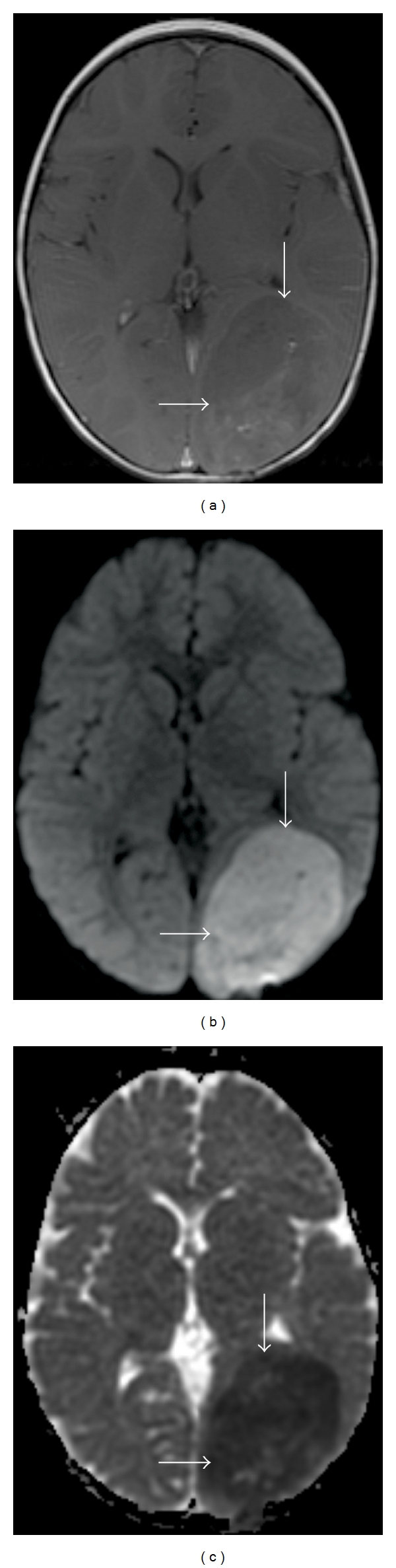
A 2-year-old female with a left parietal primitive neuroectodermal (PNET), WHO grade IV. (a) Axial T1 postcontrast. A faintly enhancing left parietal lobe mass is present (arrows). (b) DWI sequence. The mass is hyperintense on diffusion-weighted imaging. (c) ADC map. Signal hypointensity of the lesion on the ADC map (arrow) is present, consistent with restricted diffusion related to dense cell packing.

While diffusion-weighted imaging shows restricted diffusion in certain tumor types, such as epidermoid, lymphoma (Figure 4), and primitive neuroectodermal tumor (PNET) (Figure 5), the sequence has had limited success in grading brain tumors, and in distinguishing between histological subtypes. Guo et al. [9] reported a lower mean ADC value as a relative aid in differentiating primary cerebral lymphoma from high-grade astrocytoma. Rumboldt et al. [11] used ADC maps to differentiate types of childhood tumors. They found that ADC values were significantly higher in pilocytic astrocytomas than in ependymomas and medulloblastomas.

 Diffusion imaging has been described in the evaluation of cystic tumors; these tumors tend to show low signal on the DWI sequence and high signal on the ADC sequence [12–15]. However, this description may be confusing, as ring enhancing metastasis of adenocarcinoma have been reported with high signal on the DWI sequence and low signal on the ADC sequence, mimicking restricted diffusion of an abscess [16, 17]. Restricted diffusion has been described in squamous cell carcinoma metastasis, radiation necrosis [18], and glioblastomas [19]. Several studies demonstrate that the ADC sequence can help differentiate primary cerebral lymphoma (PCL) from high-grade gliomas [9, 10, 20–22], and brain metastasis [10] from PCL; however, other studies have reported overlap between these tumor types [23, 24]. The diffusion sequences should, therefore, be interpreting with caution, and in the context of reviewing the entire MR study and clinical history.

## 2. Diffusion Tensor Imaging and White Matter Tracking

Diffusion tensor imaging (DTI) represents a further development of diffusion-weighted imaging [25], investigating more than just the diffusion coefficient. DTI measures both the direction and magnitude of proton movement within the voxel for multiple dimensions of movement, using a mathematical construct, the diffusion tensor, to represent this information. Water in cerebrospinal fluid in a ventricle is essentially free to diffuse unimpeded in all directions and would be represented as a sphere. Water molecules along the axons of white matter tracts are in a highly structured environment and thus the diffusion becomes highly directional along the length of the tract [26]. Displaying this type of structured diffusion would be represented by an ellipsoid, or even multiple ellipsoids, if more than one white matter tract were interdigitating. 

Multiple ways have been devised to display these complex datasets. One of the simpler methods is the Fractional Anisotropy (FA) map, which is simply a measure of how distorted the diffusion spheroid has become at the point of measurement. A scalar value between 0 and 1 describes the degree of anisotropy or restricted movement. A value closer to 0 is assigned if the voxel is spherical, and a value near 1, if the voxel is an ellipsoid tending to a line. In other words, the value of 0 means that diffusion is isotropic, that is, it is unrestricted or free to move in all directions. A value of 1 means that diffusion occurs along a single axis, that is, not free to move in other directions. Therefore, white matter, which has diffusion ellipsoids oriented along the course of fiber tracts, has a high FA value, whereas cerebrospinal fluid has low FA values, indicative isotropic diffusion [7].

FA has been used in the study of brain neoplasms [24, 27]. However, in contrast to ADC, the relationship between FA and cellularity has not been substantiated [21]. Toh et al. [22] reported significantly decreased FA values in highly cellular PCL compared with glioblastomas, while Kinoshita et al. [28] demonstrated high FA values in the PCLs in their series. Further study is needed in this area.

**Figure 6:**
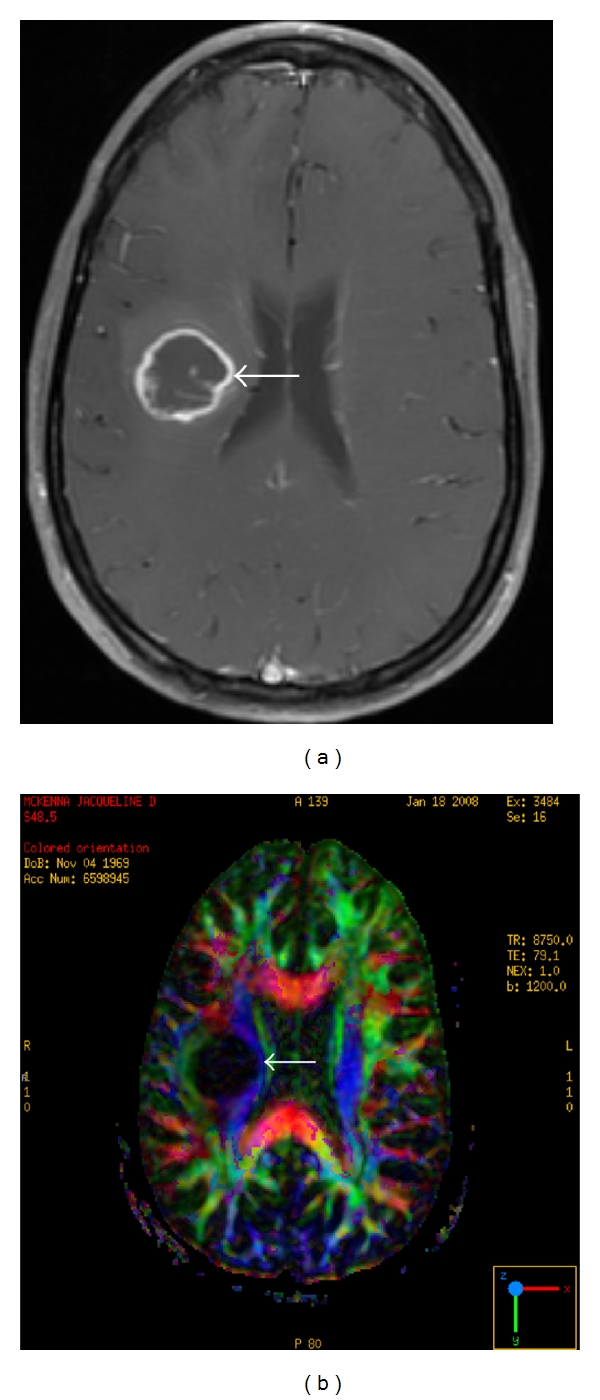
A 41-year-old woman with a right frontal lobe glioblastoma. (a) Axial T1 postcontrast. A centrally necrotic, ring enhancing mass is present in the posterior right frontal lobe (arrow). (b) Axial diffusion tensor image. The mass causes medial displacement of adjacent corticospinal tract (arrow) comparison is made to the contralateral side. Color-coded nomenclature is present in the right lower corner.

More sophisticated measures involve the use of color coding to communicate and display information about the direction of white matter tracts. Typically, one color will encode the front-to-back direction, and other will encode colors the two other cardinal directions (Figure 6). The colorful maps thus generated are very useful for portraying the complexities of white matter anatomy more faithfully than possible with FA maps alone. Given that diffusion anisotropy and white matter structure are tightly linked, it makes sense that white matter tracts could be mapped with this information [29].

Having knowledge of the dominant vectors (eigenvectors) of diffusion for a particular voxel allows one to use these as input values for probabilistic algorithms. Such an algorithm, given a seedpoint in a cerebral diffusion map, will be able to propagate itself through the space in a manner that might closely approximate the true anatomy of white matter tracts. Such methods are useful, and frequently combined with functional MR images to provide surgical guidance during surgeries near eloquent brain. These imaging methods have proven a very useful adjunct to intraoperative surgical stimulation. However, these methods have significant shortcomings when complex tracts cross through each other, and more sophisticated methods of navigating using these principles are under development [30, 31]. 

### 2.1. Application: Surgical Planning

The goal of brain tumor resection is to remove the maximal amount of tumor while preserving vital portions of brain. Diffusion tensor imaging allows mapping of white matter tracts and their relation to tumors can be demonstrated preoperatively. This can assist the neurosurgeon in planning the most appropriate approach to the tumor, maximizing the amount of tumor resected, and avoid vital structures, such as the corticospinal tract. The white matter tracts in the involved hemisphere can then be compared to the contralateral side, to determine the degree of displacement (Figure 6). Several sites in the United States have an MRI unit in the operative suite; this allows intraoperative imaging, including DTI. A recent report by Nimsky et al. describes the success of this technique in localization of shifting white matter tracts during neurosurgical procedures [32].

## 3. ADC Histogram Analysis

### 3.1. Technique

The technique of calculating the ADC coefficient for a given volume on a voxel by voxel basis for histogram analysis has been described by several authors [33–35]. Important differences exist between ADC analyses of these authors. Pope et al. [33, 34] used ADC values from regions of interest corresponding to the enhancing portion of the tumor only; they excluded regions of nonenhancing T2-weighted signal hyperintensity, which represent edema and/or infiltrative tumor [33, 34]; Murkami et al. [35] included these regions of T2 signal hyperintensity in their analysis.

### 3.2. Application: Predicting Glioblastoma Response to Bevacizumab (Avastin) Treatment and Progression-Free Survival in Bevacizumab-Treated Glioblastoma

Vascular endothelial growth factor (VEGF) and its receptors are highly expressed in glioblastomas [36], an aggressive cerebral neoplasm. VEGF is a potential mediator of cerebrovascular permeability, hypothesized to promote new blood vessel formation as well as transformation into a more aggressive phenotype [37, 38]. 

Bevacizumab is a monoclonal antibody that targets VEGF and prevents it binding to its tyrosine kinase receptors. Inhibiting the VEGF pathway improves survival in patients with glioblastoma multiforme (GBM) [39, 40]. However, the response is variable and there is no way to predict how patients will respond. Variable expression of VEGF could be related to the mixed response to bevacizumab. In regions of high cell density surrounding the necrotic areas, VEGF is upregulated [41]. Diminished enhancement and edema, apparently related to inhibition of vessel permeability by VEGF, result from treatment with bevacizumab [42]. 

Necrosis caused by therapy or tumor growth degrades cellular integrity and is thought to increase the ADC of tissue [42, 43]. This is in contradistinction to areas of high cell density, where the ADC is low, since water molecules are more restricted in their movement within cells compared to the extracellular space [44, 45]. The ADC has been used to assess brain tumor response to therapy [45, 46] and to predict survival in patients with newly diagnosed GBM [35, 47, 48].

**Figure 7:**
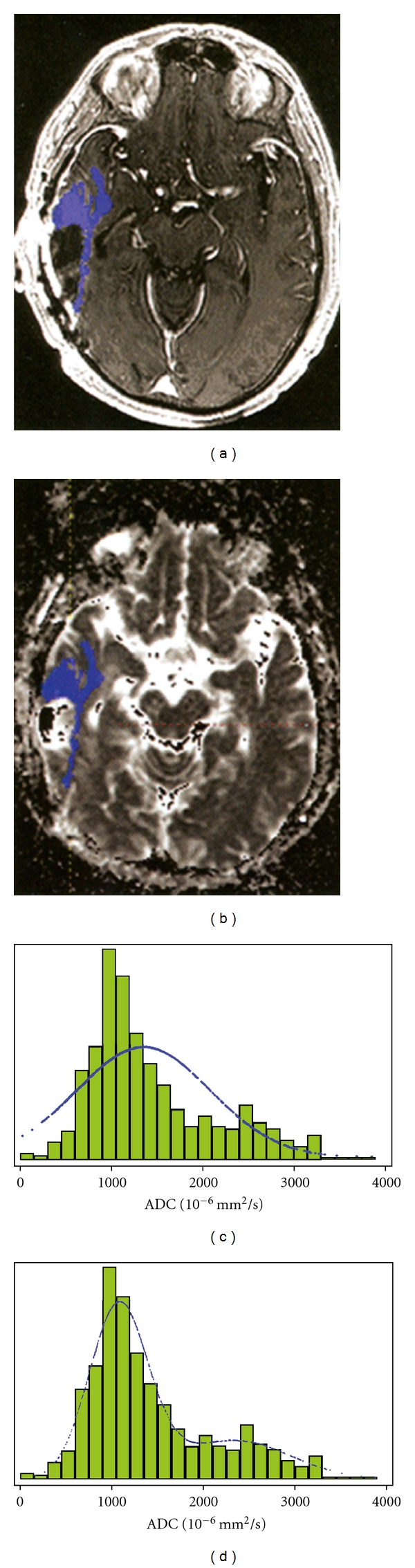
The use of ACD histograms in the analysis of cerebral malignancies. (a) Total enhancing tumor volume was segmented on axial postcontrast T1-weighted images in a 52-year-old woman with recurrent GBM and transferred to (b) the corresponding ADC map image for generation of ADC histogram (c, d). (c) A single distribution-fitted curve provided a poor fit of the data, which could be substantially improved by using (d) a two-component normal mixture model (From [33]) Reprinted with permission.

Pope et al. [33] hypothesized that bevacizumab treatment is more effective in a necrotic tumor. Utilizing the aforementioned technique, they calculated the ADC on a voxel by voxel basis for over the enhancing portion of the tumor mass and generated a histogram curve. Two peaks were present in the histogram curve analysis, and mean values were calculated for the lower (ADC_L_) and upper peaks (ADC_H_), and average ADC (Figure 7). Tumors were separated by using a cut-off for mean ADC_L_ of 1200 10^−6^ mm^2^/sec. Tumors with ADC_L_ less than 1200 10^−6^ mm^2^/sec were referred to as “low ADC tumors”, whereas tumors with mean ADC_L_ value greater or equal to 1201 10^−6^ mm^2^/sec were referred to as “high ADC tumors”. The low ADC component is thought to represent tumors with tightly packed cells and minimal edema. This is in contradistinction to the high ADC component which may represent viable and necrotic tumor cells and associated edema.

 Pope et al. [33] found that bevacizumab extends progression-free survival in patients with recurrent glioblastoma by a greater degree, if a high ADC_L_ is present in the enhancing portion of the tumor. The authors speculate that the higher ADC_L_ component might be related to intratumoral necrosis, as patients with a higher necrotic component seemed to benefit most from bevacizumab. 

In a second study, Pope et al. [34] found for patients treated initially with bevacizumab, a lower ADC_L_ was associated with longer progression-free survival compared to high ADC_L_. More research is clearly required, but there are tantalizing indications that diffusion MR might be helpful in stratifying patients into clinical risk groups and predicting the outcomes of therapies. Further indications are that patients with significantly low ADC components on their histograms may be overexpressors of O 6-methylguanine methyltransferase (MGMT), a DNA repair mechanism that imparts sensitivity to temozolomide, a standard therapeutic in brain tumor therapy [34].

## 4. Conclusion

This article presents an overview of the current state of diffusion-weighted and diffusion tensor MR imaging of the brain in patients with cancer. In acute stroke, DWI demonstrates decreased diffusion in a vascular territory affected by ischemia. Similarly, decreased diffusion is present in the center of pyogenic abscesses and aids in the MR diagnosis of a ring-enhancing cerebral mass. In addition, tumors such as lymphoma and PNET also demonstrate decreasing diffusion, adding valuable information to the radiologist when formulating a differential diagnosis of a cerebral mass lesion. There are also growing applications in differentiating tumors such as glioblastoma, primary cerebral lymphoma, and metastasis. Diffusion tensor imaging helps the neurosurgeon in planning the surgical approach to a cerebral neoplasm by demonstrating the location of white matter fiber tracts in relation to the tumor; intraoperatively fiber tract displacement can be assessed. Of great interest is the use of histogram analysis in patients with glioblastoma to stratify patients into clinical risk groups and to predict the outcomes of therapies. We anticipate that as research continues, both diffusion-weighted and diffusion tensor MR imaging will have an impact on both diagnosis as well as treatment of this patient with cerebral neoplasms.
